# Early blood biochemical changes following colostrum intake in newborn Murrah buffalo calves: an observational study

**DOI:** 10.3389/fimmu.2026.1962704

**Published:** 2026-09-28

**Authors:** Kartikey Patel, Gaurav Tripathi, Ranjeet Verma, Priyanka Singh, Shweta Yadav, Nitin Tyagi, Manoj Kumar Singh, Naresh L. Selokar

**Affiliations:** 1 Animal Biotechnology Division (ABTD), ICAR-National Dairy Research Institute (NDRI), Karnal, Haryana, India; 2 Livestock Research Centre, ICAR- ICAR-National Dairy Research Institute (NDRI), Karnal, Haryana, India

**Keywords:** buffalo, colostrum, cytokines, hematology, serum biochemistry

## Abstract

Colostrum is rich in macronutrients and bioactive components that support physiological adaptation during the neonatal period in ruminants. However, information on the early hematological, biochemical, and immunological changes to colostrum intake in buffalo calves remains limited. The present study evaluated changes in these parameters before and after colostrum intake in newborn Murrah buffalo calves (n = 20). Blood samples were collected before colostrum intake (within 30 min after birth) and after colostrum intake (12–15 h after birth). All buffalo calves had post-colostrum serum total protein (TP) concentrations >6 g/dL and serum Brix values ≥9%, which are indirect indicators of adequate transfer of passive immunity. Hematological parameters showed relatively minor changes, including a decrease in hemoglobin concentration from 15.42 ± 0.33 to 14.03 ± 0.33 g/dL. In contrast, marked increases were observed in serum biochemical parameters. Mean TP, gamma-glutamyl transferase (GGT) activity, and serum Brix values increased from 4.60 ± 0.06 g/dL, 20.14 ± 2.81 U/L, and 7.25 ± 0.12%, respectively, before colostrum intake to 7.67 ± 0.28 g/dL, 2247.58 ± 331.64 U/L, and 10.0 ± 0.3%, respectively, after colostrum intake. Cytokine profiling showed increased levels of IFN-γ, IL-1α, IL-6, IL-10, IL-17A, MIP-1α, MIP-1β, IP-10, TNF-α, and MCP-1, whereas IL-8 and VEGF-A decreased after colostrum intake. Overall, these findings provide baseline information on early blood biochemical changes following colostrum intake in neonatal Murrah buffalo calves.

## Introduction

Colostrum is essential for neonatal survival and development in ruminants. Timely colostrum intake provides immunoglobulins and diverse bioactive components, including cytokines, growth factors, vitamins, and hormones, which support passive immunity, intestinal maturation, metabolic adaptation, and early growth (1, 2). Studies in dairy cattle have documented substantial hematological and serum biochemical changes following colostrum intake (3–7). However, comparable information in buffalo calves remains scarce.

Buffaloes are important livestock species in tropical regions and have specific physiological characteristics that differ from cattle. High neonatal mortality remains a major constraint to buffalo production, with calf losses reported to reach up to 40% (8, 9. The Murrah is an economically important dairy buffalo breed, particularly in India and Brazil, and contributes substantially to milk production (10, 11). Despite its importance, early hematological, biochemical, and immunological responses to colostrum intake in Murrah buffalo calves remain poorly characterized.

Serum IgG concentration ≥10 g/L has traditionally been used as a benchmark for successful transfer of passive immunity (TPI) in neonatal cattle calves (12, 13). However, direct measurement of IgG is relatively costly and may not be routinely feasible under farm conditions. Therefore, serum total protein (TP), gamma-glutamyl transferase (GGT) levels, and serum Brix percentage (%) can be used as practical indirect indicators of TPI (14–17). In cattle, TP ≥5.5 g/dL, GGT level ≥555 IU/L, and Brix values ≥8.1% have been suggested as indicators of successful TPI (13, 14, 18). Using these cattle-based values as reference criteria, in the present study, neonatal Murrah buffalo calves that had post-colostrum serum TP values >6 g/dL and Brix values ≥9% TP were enrolled, and recorded their hematological, serum biochemical, and cytokine changes before and after colostrum intake.

## Materials and methods

### Animal management, and sample collection

All experimental procedures were conducted in accordance with the ethical standards and guidelines of the Institutional Animal Ethics Committee (IAEC) of the ICAR-National Dairy Research Institute (NDRI), Karnal, India. Animal use and procedures, including blood collection, were complied with the ARRIVE guidelines. The buffalo calves used in this investigation were born at the Livestock Research Centre, ICAR–NDRI, Karnal, India and maintained under standard farm management practices. In this study, a total of 20 healthy neonatal buffalo calves (male: 8 and female: 12) were enrolled immediately after birth. Metadata of dams and calves are provided in **Supplementary Table 1**.

Approximately 10 days before the expected date of calving, pregnant dams were moved to individual calving pens. Immediately after birth, the dam was allowed to lick the calf for approximately 10 min to facilitate maternal bonding and normal maternal behavior. Colostrum was subsequently collected by hand milking into a clean bucket and fed to the calf at 10% of the calf’s body weight. All dams included in the study had normal, uncomplicated pregnancies and natural deliveries, with no cases of dystocia or retained placenta. The first blood samples were collected immediately after birth, within 30 minutes and before colostrum feeding. The first colostrum feeding was done with in 1 hour after birth, followed by a second colostrum feeding at approximately 8 hours after birth. Both feedings were provided by bottle at 10% of the calf’s body weight. Thereafter, the calves were allowed to suckle and consume colostrum for approximately 5 mins when they reached the milking station, and then milked as per the routine management practices. Since we could not precisely control the amount of subsequent colostrum intake after the initial two feedings, thus, the second blood samples were collected at 12–15 hours. The 12–15 h post-colostrum sampling point represents an early/intermediate phase of colostrum-derived component absorption and may not reflect the stabilized status of TPI following completion of gut closure. The absence of additional sampling at 24–48 h is therefore a limitation of the present study. Future studies should include multiple post-colostrum time points to better characterize the kinetics of biochemical changes in neonatal buffalo calves.

Blood samples were collected aseptically from the jugular vein using sterile 21G needles. Samples for hematological analysis were collected in EDTA-coated vacutainers, whereas samples for serum were collected in serum-activator tubes. Serum was separated by centrifugation at 3000 rpm for 15 minutes, aliquoted, and stored at −80 °C until further analysis. As per farm records, no mortality has been reported in the selected calves, up to 3 months of age.

### Blood biochemical analysis

Collected samples were analyzed for hematological, serum biochemical, Brix, and cytokine parameters. Hematological analysis was performed within 1–2 h of blood collection using a fully automated veterinary hematology analyzer (Biovet Smart-3Dx, Biovet, India), following the manufacturer’s instructions. The erythrocyte profile included red blood cell count (RBC), hemoglobin (Hb), hematocrit (HCT), mean corpuscular volume (MCV), mean corpuscular hemoglobin (MCH), and mean corpuscular hemoglobin concentration (MCHC). The leukocyte profile included total white blood cell count (WBC), while platelet parameters included platelet count (PLT), mean platelet volume (MPV), and plateletcrit (PCT). All parameters were recorded in the units provided by the analyzer.

Serum biochemical parameters, including glucose, aspartate aminotransferase (AST), alanine aminotransferase (ALT), gamma-glutamyl transferase (GGT), alkaline phosphatase, total, direct and indirect bilirubin, total protein, albumin, globulin, albumin-to-globulin ratio (A:G), urea, blood urea nitrogen (BUN), creatinine, uric acid, total calcium, phosphorus, sodium, potassium, and chloride, were estimated by a certified veterinary diagnostic facility (Dr. Lal Path Labs, New Delhi, India; https://www.pathvets.in/) using standard analytical protocols, and provided reports were used for analysis (**Supplementary Table 2**). Serum Brix percentage was determined using a commercial digital refractometer (RX-5000i-Plus, ATAGO, Japan; measurement range: 0–100% Brix; accuracy: ± 0.010% Brix at 20 °C) with automatic temperature compensation. The instrument was calibrated to zero using distilled water before analysis. For each calf, 300 µL of thawed serum was used, and three replicate measurements were performed. The mean of the three readings was considered the final serum Brix value.

To qualitatively confirm the presence of immunoglobulins in serum following colostrum intake, SDS-PAGE was performed. Serum samples were diluted 1:100 (v/v) in triple-distilled water, and 15 µL of each diluted sample was mixed with 15 µL reducing sample loading buffer and denatured by heating. The prepared samples were loaded onto a 12% resolving polyacrylamide gel with a 4% stacking gel, along with a molecular weight protein marker. Electrophoresis was performed under standard conditions, and the gel was subsequently stained with Coomassie Brilliant Blue to visualize the separated protein bands. Bands corresponding to the approximate molecular masses of the immunoglobulin heavy chain (~55 kDa) and light chain (~25 kDa) were identified based on their migration relative to the molecular weight marker. Gel images were captured using an Azure 200 gel imager (Azure Biosystems, Dublin, California, USA).

For cytokine profiling, serum samples were pooled into three tubes, with each pool containing serum from three calves. Cytokines were quantified using a bovine cytokine magnetic bead multiplex assay kit (BCYT1–33 K-PX15, Merck Millipore, Darmstadt, Germany) according to the manufacturer’s instructions. Data were acquired using a Bio-Plex MAGPIX multiplex reader and analyzed using Bio-Plex Manager 6.0 software. The panel included pro-inflammatory cytokines [interferon-gamma (IFN-γ), interleukin-1 alpha (IL-1α), IL-1 beta (IL-1β), IL-6, IL-8, IL-17A, tumor necrosis factor-alpha (TNF-α), macrophage inflammatory protein-1 alpha (MIP-1α), macrophage inflammatory protein-1 beta (MIP-1β), and monocyte chemoattractant protein-1 (MCP-1)], anti-inflammatory cytokines [IL-4 and IL-36 receptor antagonist (IL-36RA)], and chemokines/growth factors [interferon gamma-induced protein-10 (IP-10) and vascular endothelial growth factor A (VEGF-A)].

### Statistical analysis

Statistical analyses were performed using GraphPad Prism software (version 8.0.2). Since the study involved paired measurements obtained from the same calves before and after colostrum intake, the normality differences was assessed using the Shapiro-Wilk test. Variables with normally distributed paired differences were analyzed using a two-tailed paired Student’s *t*-test, whereas variables that did not meet the assumption of normality were analyzed using the Wilcoxon signed-rank test. All statistical tests were two-sided, and a *P*-value < 0.05 was considered statistically significant. Data are presented as mean ± standard error of the mean (SEM).

## Results and discussions

In ruminants, the neonatal intestine is highly permeable immediately after birth, allowing efficient absorption of colostrum-derived macromolecules and bioactive components, including immunoglobulins, into the circulation (1). Timely colostrum intake is therefore essential for successful TPI and supporting early neonatal development. In this study, we recorded the blood biochemical changes in newborn buffalo calves during the pre- and early post-colostrum intake period (12–15 h after birth). Because direct measurement of serum IgG is not routinely feasible under farm conditions, serum TP and Brix values are commonly used as practical indirect indicators of TPI in newborn calves (14, 19). Based on established TP thresholds in cattle calves, concentrations of <5.1, 5.1–5.7, 5.8–6.1, and ≥6.1 g/dL are classified as poor, fair, good, and excellent TPI, respectively (13). Similarly, studies in bovines have reported an association between serum Brix values and serum IgG concentrations, supporting the use of Brix refractometry as an indirect measure of TPI (14). Brix values of approximately 8.1% or higher have been suggested as indicative of adequate TPI in neonatal calves (20). In the present study, all calves had post-colostrum serum TP values >6 g/dL, Brix values ≥9%, and presence of immunoglobulins (**Figure 1**), indicate successful TPI.

**Figure 1:**
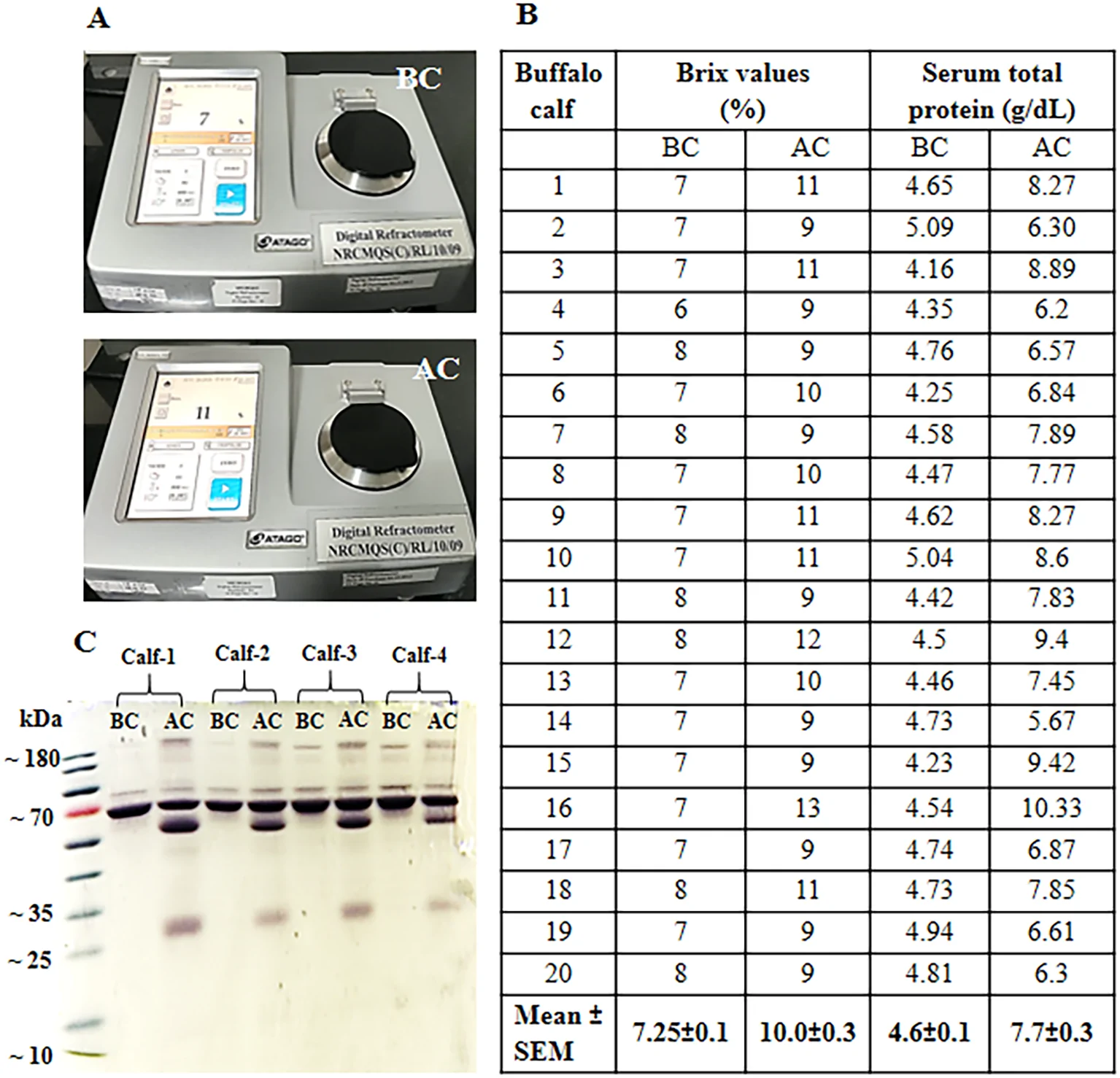
Assessment of serum samples before and after colostrum intake. **(A)** Representative images of the digital refractometer displaying serum Brix values before (BC) and after colostrum (AC) intake. **(B)** Individual serum Brix (%) and total protein (g/dL) values measured before (BC) and after colostrum (AC) intake in neonatal Murrah buffalo calves. These parameters were used as indirect indicators of transfer of passive immunity. In cattle calves, TP ≥5.5 g/dL, and Brix values ≥8.1% have been suggested as indicators of successful TPI (13, 14, 18). **(C)** Representative stained SDS-PAGE gel showing two prominent bands at approximately 55 kDa and 25 kDa, corresponding to the heavy and light chains, respectively, of IgG in post-colostrum (AC) serum samples.

The hematological parameters measured in this study are presented in **Figure 2**. The observed changes were consistent with the normal physiological adaptation of newborn calves during the early neonatal period. Similar hematological changes have also been reported in neonatal cattle calves (3, 21). Among the hematological parameters, hemoglobin (Hb) exhibited the least individual variation at birth, suggesting that it may serve as a relatively stable parameter of neonatal health. Hb concentration marginally declined from 15.42 ± 0.33 g/dL before colostrum intake to 14.03 ± 0.33 g/dL after colostrum intake (approximately 9.1%). Notably, the Hb concentrations observed in the present study were higher than those reported previously in buffalo calves, where mean Hb values of approximately 12 g/dL (22). This discrepancy is likely attributable to differences in breed, maternal nutrition, neonatal management, environmental conditions, and, importantly, the timing of blood sampling. For example, Patel et al. (22) evaluated hematological parameters in one-year-old Banni buffalo, whereas the present study examined newborn Murrah buffalo calves within the first 15 hours after birth.

**Figure 2:**
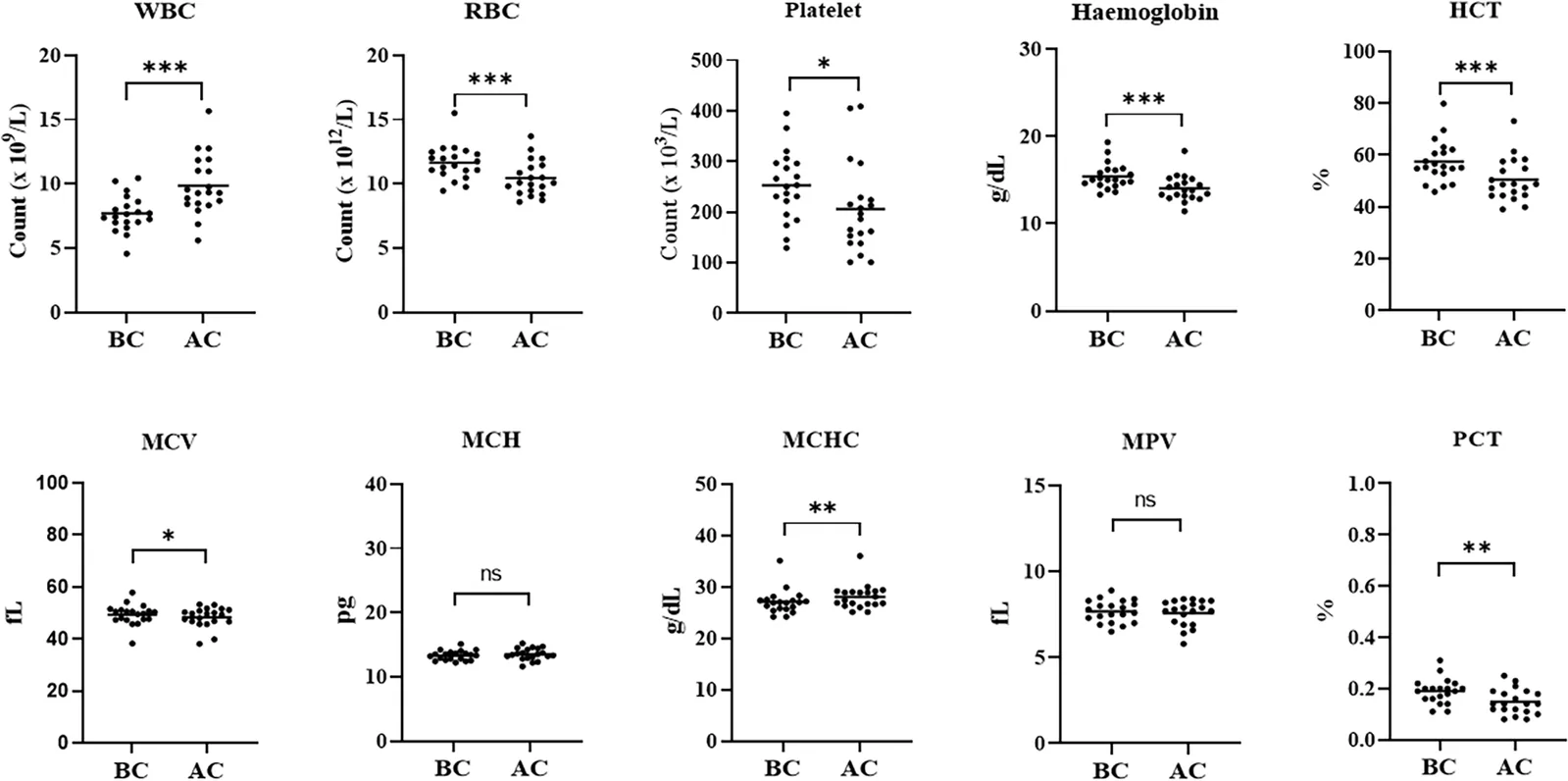
Hematological parameters in new born buffalo calves. BC, Before colostrum feeding; AC, After colostrum feeding; RBC, Red blood cell count; WBC, white blood cell count; MCV, mean corpuscular volume; MCH, mean corpuscular haemoglobin; MCHC, mean corpuscular hemoglobin concentration; HCT, Hematocrit; MPV, mean platelet volume- MPV; PCT, plateletcrit, p < 0.05: *, p < 0.01: **, p < 0.001: ***, ns, not significant.

Serum biochemistry analysis recorded marked changes between pre- and post-colostrum intake period (**Figure 3**). Among the different serum parameters, TP, GGT, and % Brix, are widely accepted as reliable indirect indicators of successful TPI (13, 14, 23, 24). These indicators exhibit minimal individual biological variation before colostrum intake but increase markedly following colostrum intake (1, 23–25). In the present study, mean serum TP, GGT, and % Brix values increased from 4.60 ± 0.06 g/dL, 20.14 ± 2.81 U/L, and 7.25 ± 0.12% pre-colostrum intake period to 7.67 ± 0.28 g/dL, 2247.58 ± 331.64 U/L, and 10.0± 0.3%, respectively, post-colostrum intake period. Similar increases have been reported previously in buffalo calves (19, 25). In cattle, successful TPI is generally associated with serum TP concentrations exceeding 5.5 g/dL, GGT level > 800 U/L, and > 8.1% Brix after post-colostrum intake period (4, 6, 14). However, the present study and previous observation found that buffalo calves typically demonstrate serum TP concentrations above 6 g/dL, GGT level above 1,500, and % Brix above 9 (19, 25) post colostrum intake. These higher concentrations likely reflect species-specific differences in colostrum composition, immunoglobulin content, enzyme levels, and neonatal physiology.

**Figure 3:**
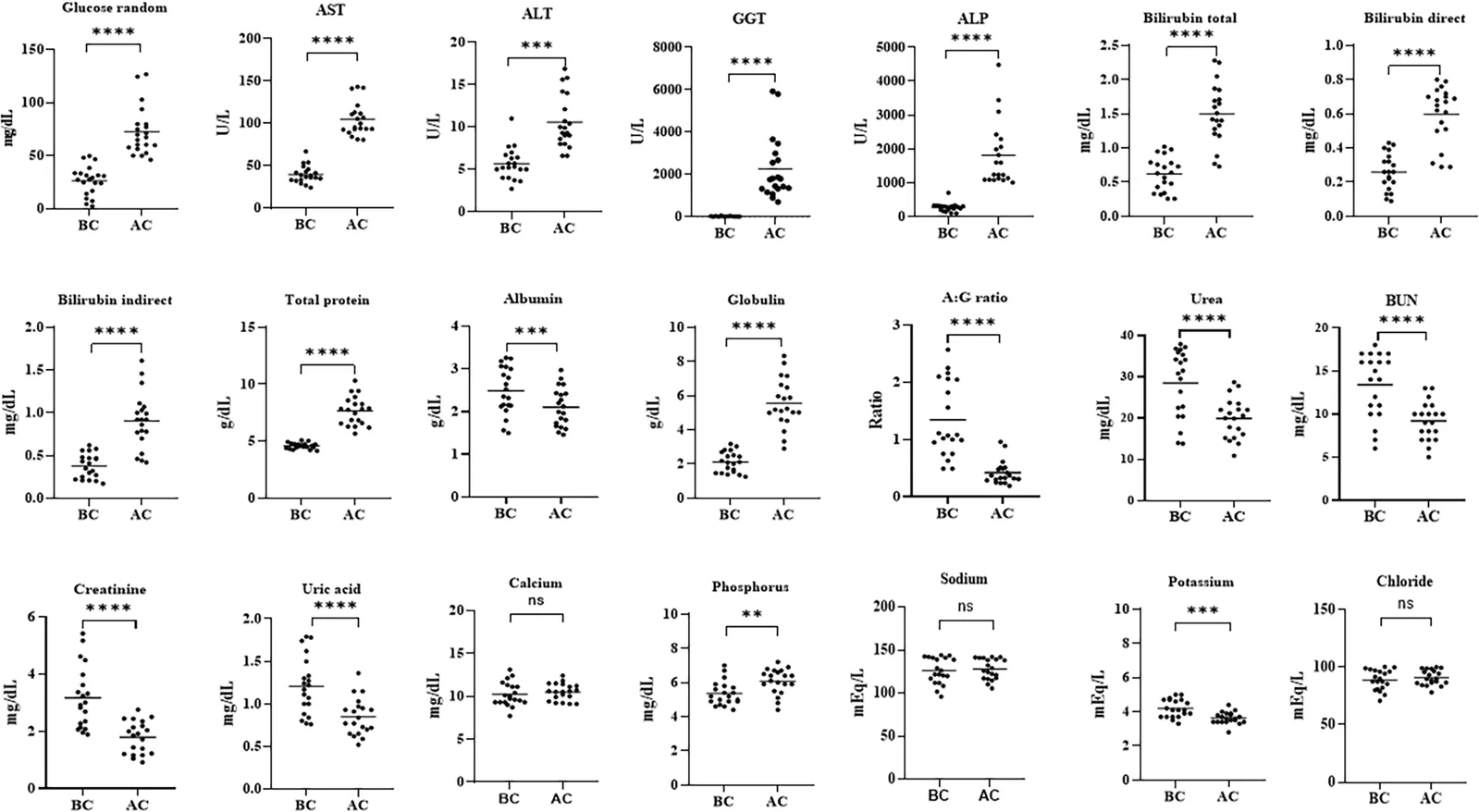
Serum biochemical parameters in new born buffalo calves. BC, Before colostrum feeding; AC, After colostrum feeding. AST, aspartate aminotransferase; ALT, alanine aminotransferase; ALP, alkaline phosphatase; GGT, gamma-glutamyl transferase; A:G, stands albumin-to-globulin ratio (A:G); BUN, blood urea nitrogen (BUN). p < 0.01: **, p < 0.001: ***, p < 0.0001: ****, ns: not significant.

In this study, SDS-PAGE was performed to confirm the presence of immunoglobulins in post-colostrum serum samples. Two prominent bands corresponding to approximately 55 kDa and 25 kDa, representing the heavy and light chains of IgG, respectively, were observed (**Figure 1**), supporting the presence of IgG in the serum samples following colostrum intake. However, because serum IgG was not quantified as a gold standard in this study, individual TPI success or failure (FPT) could not be definitively verified; without an FPT control cohort, the present findings are restricted to baseline physiological changes following colostrum intake rather than a validated diagnostic assessment of TPI. Thus, there is a need to validate recorded serum biochemical values in larger populations encompassing different buffalo breeds, maternal parity, colostrum quality, management systems, and geographical regions. In addition to GGT enzyme level, other serum enzymes such as AST, ALT, and ALP were also exhibited relatively low individual variations in pre-colostrum calf serum and significantly increased following colostrum intake (**Figure 3**; **Supplementary Table 3**). However, unlike GGT, their post-colostrum increase is not fully studied. Thus, further studies are needed to determine their relationship with colostrum presence, intestinal absorption, and TPI.

Studies in cattle have reported changes in serum cytokines following colostrum intake and their possible association with early neonatal health (26–28). However, there is no information on cytokine changes following colostrum intake in buffalo calves. In the present study, 15 serum cytokines were evaluated before and after colostrum intake (**Figure 4**). The levels of IFN-γ, IL-1α, IL-6, IL-10, IL-17A, MIP-1α, MIP-1β, IP-10, MCP-1, and TNF-α increased after colostrum intake, whereas IL-8 and VEGF-A showed a decrease. These changes indicate an overall alteration in the serum cytokine profile during the early period following colostrum intake. However, the cytokine findings should be considered preliminary and exploratory because serum samples were pooled before analysis, and individual animal variation could not be assessed. Therefore, these results cannot be used to establish definitive cytokine values or individual-level responses.

**Figure 4:**
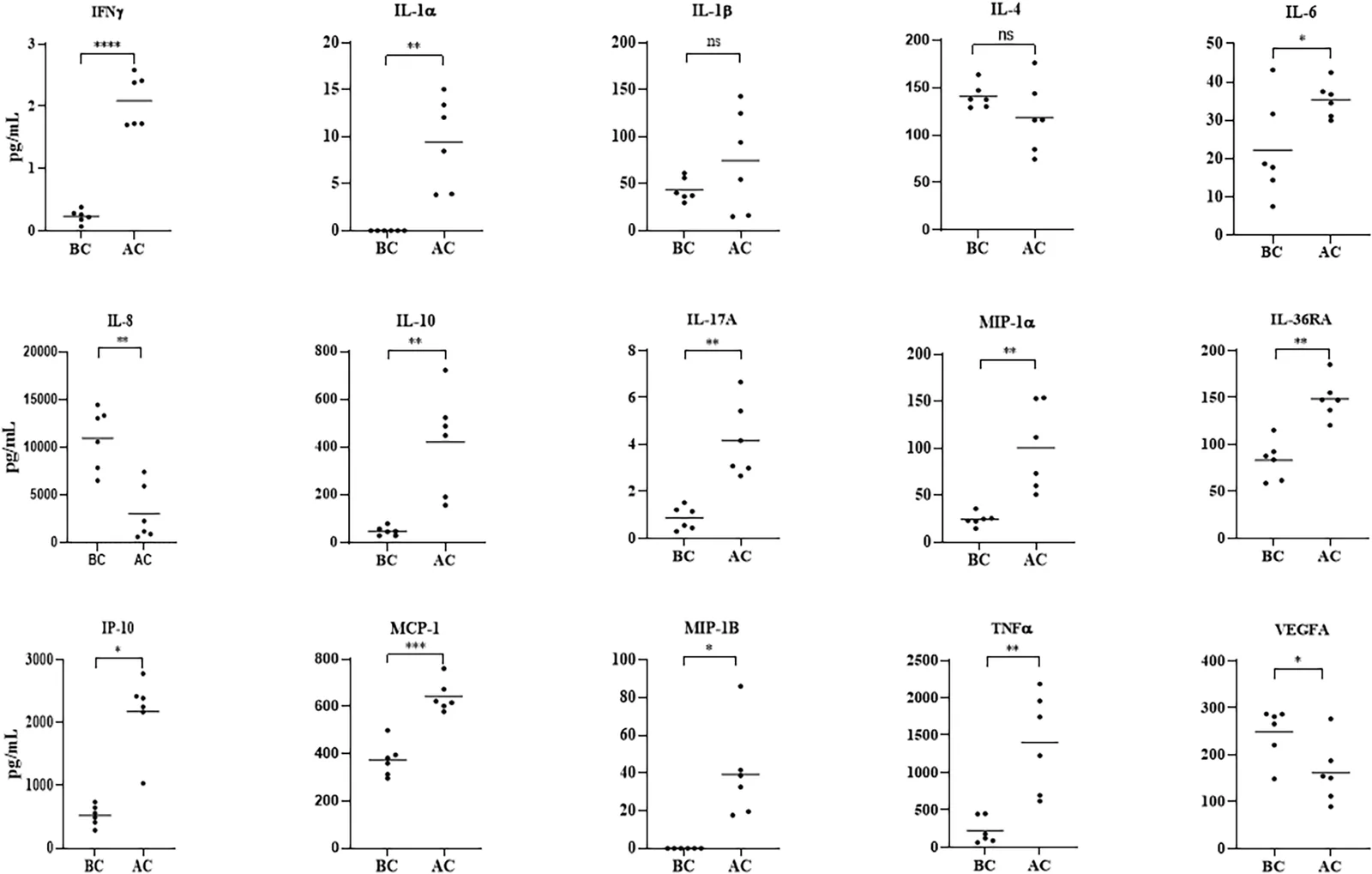
Serum cytokines levels in new born buffalo calves. BC, Before colostrum feeding; AC, After colostrum feeding. p < 0.05: *, p < 0.01: **, p < 0.001: ***, p < 0.0001: ****, ns: not significant.

In addition, an important limitation of the present study is a relatively small cohort of 20 buffalo calves. Although paired t-tests were appropriate for comparing the same animals before and after colostrum intake, no formal adjustment for multiple comparisons was applied across the different outcomes. Therefore, the statistical findings should be interpreted as exploratory rather than confirmatory, and the possibility of an increased Type I error due to multiple testing cannot be excluded. Thus, further studies using larger sample sizes, and diverse management conditions are needed to determine definitive changes following colostrum intake in buffalo calves.

In conclusion, this study provides baseline information on blood hematological, biochemical, and cytokine changes during the pre- and post-colostrum intake period in newborn buffalo calves. However, the standard reference values for this species should be generated in larger populations, and diverse management, nutritional, and environmental conditions.

## Data Availability

The original contributions presented in the study are included in the article/**Supplementary Material**. Further inquiries can be directed to the corresponding author.
