# Supplementary material for: Early blood biochemical changes following colostrum intake in newborn Murrah buffalo calves: an observational study
Source: Front Immunol. 2026 Sep 28;17:1962704. doi: 10.3389/fimmu.2026.1962704 (PMC13654749; doi:10.3389/fimmu.2026.1962704)
Supplement: Supplementary file 1 [file DataSheet1.pdf]

Supplementary table 1: Metadata of dams and calves enrolled in this study

Dam no.	Gestation length (Days)	Parity	Calving date, and time	Calf code	Sex	Birth weight (Kg)	Observation as on 24th Aug, 2026
MU-8053	310	1	14-08-24	1.	Female	33	Live at farm
MU-8036	318	1	17-08-24	2.	Female	40	Live at farm
MU-7789	302	2	17-08-24	3.	Male	26	Died on 15-01-25
MU-8321	304	1	04-09-24	4.	Female	39	Live at farm
MU-8072	310	1	15-09-24	5.	Male	28	Live at farm
MU-8031	307	1	15-09-24	6.	Female	30	Live at farm
MU-6905	301	3	28-09-24	7.	Male	30	Reserve for bull selection
MU-8173	310	1	28-09-24	8.	Female	29	Live at farm
MU-8082	298	1	01-10-24	9.	Male	37	Live at farm
MU-7871	305	2	02-10-24	10.	Male	28	Sold on 15-10-25
MU-6610	301	3	11-10-24	11.	Male	32	Sold on 15-10-25
MU-8168	297	1	16-10-24	12.	Female	35	Live at farm
MU-8144	309	1	20-10-24	13.	Female	31	Live at farm
MU-7130	314	3	21-10-24	14.	Female	33	Live at farm
MU-8145	299	1	24-10-24	15.	Female	30	Live at farm
MU-8174	297	1	06-11-24	16.	Male	32	Sold on 15-10-25
MU-7965	310	2	06-11-24	17.	Female	29	Live at farm
MU-8007	308	2	15-11-24	18.	Male	31	Sold on 15-10-25
MU-7844	306	2	20-11-24	19.	Female	34	Live at farm
MU-7608	314	3	21-11-24	20.	Female	30	Sold on 15-10-25
