# Supplementary material for: Early blood biochemical changes following colostrum intake in newborn Murrah buffalo calves: an observational study
Source: Front Immunol. 2026 Sep 28;17:1962704. doi: 10.3389/fimmu.2026.1962704 (PMC13654749; doi:10.3389/fimmu.2026.1962704)
Supplement: Supplementary file 2 [file DataSheet2.pdf]

PathVets™
A venture of Dr Lal PathLabs

Name	: Buffalo MU.8145.BC .	Age	: 1 Day
Lab No.	: 394064728	Gender	: Unknown
Ref By	: SELF	Reported	: 16/03/2025 12:57:31 PM
Collected	: 13/03/2025 06:05:00 PM	Report Status	: Interim
A/c Status	: P		
Collected At	: PATHVETS HARYANA<br>SCS-3, Sector-14, Main MKT, SONIPAT - 131001	Processed At	: PathVets C R Park<br>GROUND FLOOR (GF), PLOT NO. 50, POCKET NO. 40, CHITTARANJAN PARK, NEW DELHI - 110019
Id	:		

Test Report

Test Name	Results	Units	Bio. Ref. Interval
**Glucose Random**	**29.4**	mg/dL	45 - 75

Customer Care No.:
+91 11 49058406, 49058407

If tests resuts are alarming or unexpected, client is advised to contact the laboratory immediately for possible remedial action.

**Name**	**: Buffalo MU.8145.BC .**	**Age**	**: 1 Day**
**Lab No.**	**: 394064728**	**Gender**	**: Unknown**
**Ref By**	**: SELF**	**Reported**	**: 16/03/2025 12:57:31 PM**
**Collected**	**: 13/03/2025 06:05:00 PM**	**Report Status**	**: Interim**
**A/c Status**	**: P**		
**Collected At**	**: PATHVETS HARYANA**<br>**SCS-3, Sector-14, Main MKT, SONIPAT - 131001**	**Processed At**	**: PathVets C R Park**<br>**GROUND FLOOR (GF), PLOT NO. 50, POCKET NO. 40, CHITTARANJAN PARK, NEW DELHI - 110019**
**Id**	**:**		

Test Report

Test Name	Results	Units	Bio. Ref. Interval
**LIVER & KIDNEY PANEL, SERUM**			
**AST (SGOT)**<br>(IFCC)	**53.90**	U/L	78 - 132
**ALT (SGPT)**<br>(IFCC)	**7.80**	U/L	11 - 40
**GGTP**	**59.60**	U/L	6.1 - 17.4
**Alkaline Phosphatase**<br>(IFCC)	**706.30**	U/L	26 - 78
**Bilirubin Total**<br>(DPD)	**0.72**	mg/dL	0.01 - 0.5
**Bilirubin Direct**<br>(DPD)	0.26	mg/dL	
**Bilirubin Indirect**<br>(Calculated)	**0.46**	mg/dL	0.0 - 0.03
**Total Protein**<br>(Spectrophotometry)	**5.04**	g/dL	6.74 - 7.46
**Albumin**<br>(BCG)	**2.52**	g/dL	3.03 - 3.55
**Globulin**<br>(Calculated)	2.52	g/dL	
**A : G Ratio**	**1.00**		0.84 - 0.94
**Urea**<br>(Urease UV)	**31.50**	mg/dL	42.88 - 64.26
**BUN**<br>(Calculated)	**15**	mg/dL	20 - 30
**CREATININE**<br>(Compensated Jaffes method, IDMS traceable)	5.42	mg/dL	
**Uric acid**<br>(Uricase)	1.62	mg/dL	0 - 2
**Calcium, Total**<br>(Arsenazo III)	11.1	mg/dL	9.7 - 12.4
**Phosphorus**<br>(Molybdate UV)	5.6	mg/dL	4.3-7.8
**Sodium**<br>(Indirect ISE)	**129.1**	mEq/L	132 - 152

Customer Care No.:
+91 11 49058406, 49058407

If tests resuts are alarming or unexpected, client is advised to contact the laboratory immediately for possible remedial action.

**Name**	**: Buffalo MU.8145.BC .**	**Age**	**: 1 Day**
**Lab No.**	**: 394064728**	**Gender**	**: Unknown**
**Ref By**	**: SELF**	**Reported**	**: 16/03/2025 12:57:31 PM**
**Collected**	**: 13/03/2025 06:05:00 PM**	**Report Status**	**: Interim**
**A/c Status**	**: P**		
**Collected At**	**: PATHVETS HARYANA**<br>**SCS-3, Sector-14, Main MKT, SONIPAT - 131001**	**Processed At**	**: PathVets C R Park**<br>**GROUND FLOOR (GF), PLOT NO. 50, POCKET NO. 40, CHITTARANJAN PARK, NEW DELHI - 110019**
**Id**	**:**		

Test Report

**LIVER & KIDNEY PANEL, SERUM**			
**Potassium**<br>(Indirect ISE)	4.1	mEq/L	3.9 - 5.8
**Chloride**<br>(Indirect ISE)	**90.1**	mEq/L	97 - 111

Result/s to follow:
IMMUNOGLOBULIN IgA(PV) (PV133)
IMMUNOGLOBULIN IgG(PV) (PV134)
IMMUNOGLOBULIN IgM (PV) (PV132)
LIPID PROFILE, BASIC

Dr Gulshan Kumar Singh
MVSC Veterinary Pathologist

------------------ End Of Report ------------------

Customer Care No.:
+91 11 49058406, 49058407

If tests resuts are alarming or unexpected, client is advised to contact the laboratory immediately for possible remedial action.

IMPORTANT INSTRUCTIONS:

- ★ Test results released pertaining to the specimen submitted.
- ★ All test results are dependent on the quality of the sample received by the Laboratory.
- ★ Laboratory investigations are only a tool to fecilitate arrival of a diagnosis and should be clinically correlated by the Referring Veterinarian.
- ★ Sample repeats are accepted on request of Referring Veterinarian with in 7 days post reporting.
- ★ Report delivery may be delayed due to unforseen circumstances. Inconvenience is regretted.
- ★ Test result may show inter-laboratory variations.
- ★ The courts/ forum at Delhi shall have an exclusive jurisdiction on all disputes/ claims concerning the test(s) and or results of the test(s).
- ★ Test results are not valid for vetro-legal purpose.

Contact customer care number:

+91 11 49058406, 49058407 for all queries related to rest of the results.

**Name**	: **Buffalo MU.8145.AC .**	**Age**	: **1 Day**
**Lab No.**	: **394064719**	**Gender**	: **Unknown**
**Ref By**	: **SELF**	**Reported**	: **16/03/2025 12:58:37 PM**
**Collected**	: **13/03/2025 06:14:00 PM**	**Report Status**	: **Interim**
**A/c Status**	: **P**		
**Collected At**	: **PATHVETS HARYANA**<br>**SCS-3, Sector-14, Main MKT, SONIPAT - 131001**	**Processed At**	: **PathVets C R Park**<br>**GROUND FLOOR (GF), PLOT NO. 50, POCKET NO. 40, CHITTARANJAN PARK, NEW DELHI - 110019**
**Id**	:		

Test Report

Test Name	Results	Units	Bio. Ref. Interval
**Glucose Random**	58.0	mg/dL	45 - 75

Customer Care No.:
+91 11 49058406, 49058407

If tests resuts are alarming or unexpected, client is advised to contact the laboratory immediately for possible remedial action.

Name	: Buffalo MU.8145.AC .	Age	: 1 Day
Lab No.	: 394064719	Gender	: Unknown
Ref By	: SELF	Reported	: 16/03/2025 12:59:11 PM
Collected	: 13/03/2025 06:14:00 PM	Report Status	: Interim
A/c Status	: P		
Collected At	: PATHVETS HARYANA<br>SCS-3, Sector-14, Main MKT, SONIPAT - 131001	Processed At	: PathVets C R Park<br>GROUND FLOOR (GF), PLOT NO. 50, POCKET NO. 40, CHITTARANJAN PARK, NEW DELHI - 110019
Id	:		

Test Report

Test Name	Results	Units	Bio. Ref. Interval
**LIVER & KIDNEY PANEL, SERUM**			
**AST (SGOT)**<br>(IFCC)	121.00	U/L	78 - 132
**ALT (SGPT)**<br>(IFCC)	16.90	U/L	11 - 40
**GGTP**	**3655.00**	U/L	6.1 - 17.4
**Alkaline Phosphatase**<br>(IFCC)	**4484.70**	U/L	26 - 78
**Bilirubin Total**<br>(DPD)	**1.40**	mg/dL	0.01 - 0.5
**Bilirubin Direct**<br>(DPD)	0.70	mg/dL	
**Bilirubin Indirect**<br>(Calculated)	**0.70**	mg/dL	0.0 - 0.03
**Total Protein**<br>(Spectrophotometry)	**8.60**	g/dL	6.74 - 7.46
**Albumin**<br>(BCG)	**2.12**	g/dL	3.03 - 3.55
**Globulin**<br>(Calculated)	6.48	g/dL	
**A : G Ratio**	**0.33**		0.84 - 0.94
**Urea**<br>(Urease UV)	**27.80**	mg/dL	42.88 - 64.26
**BUN**<br>(Calculated)	**13**	mg/dL	20 - 30
**CREATININE**<br>(Compensated Jaffes method, IDMS traceable)	2.76	mg/dL	
**Uric acid**<br>(Uricase)	0.77	mg/dL	0 - 2
**Calcium, Total**<br>(Arsenazo III)	10.6	mg/dL	9.7 - 12.4
**Phosphorus**<br>(Molybdate UV)	5.9	mg/dL	4.3-7.8
**Sodium**<br>(Indirect ISE)	**129.0**	mEq/L	132 - 152

Customer Care No.:
+91 11 49058406, 49058407
If tests resuts are alarming or unexpected, client is advised to contact the laboratory immediately for possible remedial action.

**Name**	: **Buffalo MU.8145.AC .**		**Age**	: **1 Day**
**Lab No.**	: **394064719**		**Gender**	: **Unknown**
**Ref By**	: **SELF**		**Reported**	: **16/03/2025 12:59:11 PM**
**Collected**	: **13/03/2025 06:14:00 PM**		**Report Status**	: **Interim**
**A/c Status**	: **P**			
**Collected At**	: **PATHVETS HARYANA**<br>**SCS-3, Sector-14, Main MKT, SONIPAT - 131001**		**Processed At**	: **PathVets C R Park**<br>**GROUND FLOOR (GF), PLOT NO. 50, POCKET NO. 40, CHITTARANJAN PARK, NEW DELHI - 110019**
**Id**	:			

Test Report

**LIVER & KIDNEY PANEL, SERUM**			
**Potassium**<br>(Indirect ISE)	**3.4**	mEq/L	3.9 - 5.8
**Chloride**<br>(Indirect ISE)	**88.6**	mEq/L	97 - 111

Result/s to follow:
IMMUNOGLOBULIN IgA(PV) (PV133)
IMMUNOGLOBULIN IgG(PV) (PV134)
IMMUNOGLOBULIN IgM (PV) (PV132)
LIPID PROFILE, BASIC

Dr Gulshan Kumar Singh
MVSC Veterinary Pathologist

------------------ End Of Report ------------------

Customer Care No.:
+91 11 49058406, 49058407

If tests resuts are alarming or unexpected, client is advised to contact the laboratory immediately for possible remedial action.

IMPORTANT INSTRUCTIONS:

- Test results released pertaining to the specimen submitted.
- All test results are dependent on the quality of the sample received by the Laboratory.
- Laboratory investigations are only a tool to fecilitate arrival of a diagnosis and should be clinically correlated by the Referring Veterinarian.
- Sample repeats are accepted on request of Referring Veterinarian with in 7 days post reporting.
- Report delivery may be delayed due to unforseen circumstances. Inconvenience is regretted.
- Test result may show inter-laboratory variations.
- The courts/ forum at Delhi shall have an exclusive jurisdiction on all disputes/ claims concerning the test(s) and or results of the test(s).
- Test results are not valid for vetro-legal purpose.

Contact customer care number:

+91 11 49058406, 49058407 for all queries related to rest of the results.
