# Supplementary material for: Early blood biochemical changes following colostrum intake in newborn Murrah buffalo calves: an observational study
Source: Front Immunol. 2026 Sep 28;17:1962704. doi: 10.3389/fimmu.2026.1962704 (PMC13654749; doi:10.3389/fimmu.2026.1962704)
Supplement: Supplementary file 3 [file DataSheet3.pdf]

Supplementary Table 3. Serum biochemical profiling of neonatal Murrah buffalo calves (n=20). Data is provided as mean along with SEM, minimum and maximum data values.

	**Before colostrum (BC)**			**After colostrum (AC)**		
	**Mean**	**SEM**	**Min-Max**	**Mean**	**SEM**	**Min-Max**
Glucose (Random)	26.35	3.13	2.4-49.9	73.67	5.15	46.3-127.1
AST (SGOT) (U/L)	39.64	2.30	24-66.9	104.53	4.35	80.5-143.1
ALT (SGPT) (U/L)	5.65	0.41	2.7-11	10.58	0.70	6.6-16.9
GGT (U/L)	20.14	2.81	7.9-59.6	2247.58	331.64	696.5-5920
Alkaline Phosphatase (U/L)	276.09	28.28	97.5-706.3	1809.00	210.87	1009.4-4484.7
Total Bilirubin (mg/dL)	0.62	0.06	0.26-1.02	1.50	0.10	0.73-2.28
Direct Bilirubin (mg/dL)	0.26	0.02	0.09-0.43	0.60	0.04	0.29-0.80
Indirect Bilirubin (mg/dL)	0.38	0.03	0.17-0.62	0.91	0.07	0.42-1.61
Total Protein (g/dL)	4.60	0.06	4.16-5.09	7.67	0.28	5.67-10.33
Albumin (g/dL)	2.49	0.12	1.5-3.26	2.10	0.10	1.46-2.97
Globulin (g/dL)	2.12	0.14	1.26-3.18	5.57	0.32	2.9-8.34
A:G Ratio	1.35	0.15	0.49-2.57	0.42	0.05	0.19-0.96
Urea (mg/dL)	28.50	1.84	13.8-37.9	19.96	1.08	10.9-28.7
BUN (mg/dL)	13.40	0.83	6-18	9.20	0.50	5-13
Creatinine (mg/dL)	3.17	0.24	1.89-5.42	1.80	0.13	0.92-2.76
Uric Acid (mg/dL)	1.21	0.08	0.76-1.79	0.85	0.05	0.52-1.36
Total Calcium (mg/dL)	10.23	0.31	7.7-13.1	10.50	0.22	9.1-12.4
Phosphorus (mg/dL)	5.36	0.16	4.4-7.0	6.08	0.16	4.4-7.2
Sodium (mmol/L)	126.00	3.40	95.9-144.3	128.03	2.68	105.6-142.1
Potassium (mmol/L)	4.20	0.12	3.3-5.0	3.63	0.08	2.8-4.4
Chloride (mmol/L)	88.98	1.96	70.8-100.3	91.08	1.47	78.2-100.1
